# Biofabrication of HepG2 Cells-Laden 3D Structures Using Nanocellulose-Reinforced Gelatin-Based Hydrogel Bioinks: Materials Characterization, Cell Viability Assessment, and Metabolomic Analysis

**DOI:** 10.1021/acsbiomaterials.4c02148

**Published:** 2025-04-17

**Authors:** Nicole S. Lameirinhas, Maria C. Teixeira, João P. F. Carvalho, Bruno F. A. Valente, Jorge L. Luís, Iola F. Duarte, Ricardo J. B. Pinto, Helena Oliveira, José M. Oliveira, Armando J. D. Silvestre, Carla Vilela, Carmen S. R. Freire

**Affiliations:** † CICECO—Aveiro Institute of Materials, Department of Chemistry, 56062 University of Aveiro, Aveiro 3810-193, Portugal; ‡ EMaRT Group—Emerging: Materials, Research, Technology, School of Design, Management and Production Technologies Northern Aveiro, University of Aveiro, 3720-509 Oliveira de Azeméis, Portugal; § LAQV-REQUIMTE, University of Aveiro, 3810-193 Aveiro, Portugal; ∥ CESAM—Centre for Environmental and Marine Studies, Department of Biology, University of Aveiro, 3810-193 Aveiro, Portugal

**Keywords:** 3D bioprinting, gelatin, genipin, hydrogel bioinks, metabolomics, nanofibrillated cellulose

## Abstract

The successful replication of the intricate architecture of human tissues remains a major challenge in the biomedical area. Three-dimensional (3D) bioprinting has emerged as a promising approach for the biofabrication of living tissue analogues, taking advantage of the use of adequate bioinks and printing methodologies. Here, a hydrogel bioink based on gelatin (Gel) and nanofibrillated cellulose (NFC), cross-linked with genipin, was developed for the 3D extrusion-based bioprinting of hepatocarcinoma cells (HepG2). This formulation combines the biological characteristics of Gel with the exceptional mechanical and rheological attributes of NFC. Gel/NFC ink formulations with different Gel/NFC mass compositions, viz., 90:10, 80:20, 70:30, and 60:40, were prepared and characterized. The corresponding cross-linked hydrogels were obtained using 1.5% (w/w) genipin as the cross-linking agent. The rheological and mechanical performances of the inks were enhanced by the addition of NFC, as evidenced by the rise in the yield stress from 70.9 ± 28.6 to 627.9 ± 74.8 Pa, compressive stress at 80% strain from 0.5 ± 0.1 to 1.5 ± 0.2 MPa, and Young’s modulus from 4.7 ± 0.9 to 12.1 ± 1.1 MPa, for 90:10 and 60:40 inks, respectively. Moreover, higher NFC contents translated into 3D structures with better shape fidelity and the possibility of printing more intricate structures. These hydrogels were noncytotoxic toward HepG2 cells for up to 48 h, with cell viabilities consistently above 80%. The ink 70:30 was loaded with HepG2 cells (2 × 10^6^ cells mL^–1^) and bioprinted. Cell viability remained elevated (90 ± 4%) until day 14 postbioprinting, with cells maintaining their metabolic activity shown by ^1^H NMR metabolomics, proving the enormous potential of Gel/NFC-based bioinks for bioprinting HepG2 cells without jeopardizing their viability and metabolism.

## Graphical abstract

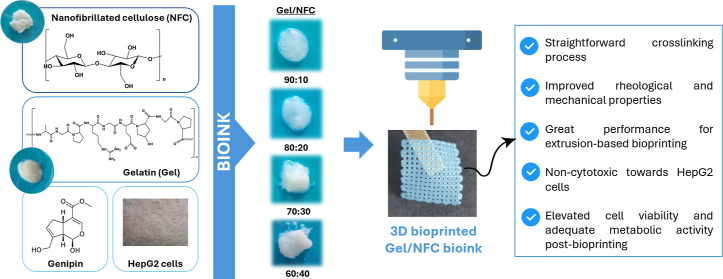

## 1 Introduction

In recent years, an increasing need has been witnessed for in vitro models that successfully mimic the cellular environment, avoiding the use of animal testing in the early stages of biomedical research toward more accurate drug screening, disease research, and design of scaffolds for tissue engineering. Two-dimensional (2D) cell cultures fail to replicate the complexity of biological systems, leading to unreliable results as cells cultured in monolayer typically behave differently from those in real tissues.^1,2^ On the other hand, three-dimensional (3D) cell cultures (e.g., hydrogels and spheroids) have appeared as an excellent option to overcome the limitations of 2D models. 3D bioprinting, the biological branch of additive manufacturing, has also gained ground as a promising strategy for the biofabrication of 3D cell cultures.^3,4^ In this technique, bioinks composed of biomaterials, living cells, and other biological compounds (e.g., growth factors) are deposited in a layer-by-layer fashion and in a defined spatial pattern using computer-aided design (CAD) to form 3D living tissue analogues.^3,4^ Indeed, this technology has already been investigated for the biofabrication of living tissues for regenerative medicine and tissue engineering,^5,6^ the development of drug delivery systems,^7,8^ and as 3D platforms for disease research, particularly in cancer research,^9^ including breast cancer,^10,11^ glioblastoma,^12,13^ melanoma,^14^ and hepatocellular carcinoma.^15,16^ With such high-end demands, the search for advanced bioinks that can be translated into 3D bioprinted structures with mechanical and morphological properties similar to native tissues is an important area of research.^17,18^ The biochemical, biological, rheological, and mechanical properties of the bioinks are critical and determine the success of the 3D bioprinting process.^19^ Hydrogels are the most commonly used type of bioinks due to their similarity to the cellular microenvironment and their ability to provide the required mechanical properties to support cell growth and proliferation.^20^ Hydrogels are typically obtained by the cross-linking of synthetic or natural polymers. Due to their inherent biocompatibility and specific biological roles, hydrogels based on polysaccharides (e.g., chitosan, alginate, and cellulose) and proteins (e.g., collagen and gelatin) are particularly attractive for these ventures.^20^ For instance, proteins are immensely valuable in various biomedical applications,^21^ including in the search for novel bioinks for 3D bioprinting applications,^22,23^ because they are abundant, biocompatible, and biodegradable. Among the myriad of available proteins, gelatin (Gel) has been extensively explored in this realm.^24^ Gelatin is a water-soluble protein obtained by the partial hydrolysis of collagen, the main fibrous protein of bones, cartilage, and skin.^25,26^ This protein promotes cell adhesion and proliferation as it retains the tripeptide arginine–glycine–aspartate (RGD) sequence of collagen. Moreover, gelatin is biocompatible and biodegradable without producing harmful byproducts and may easily be chemically functionalized and processed into hydrogels by thermal treatment, making it a versatile biomaterial for various 3D bioprinting applications.^24,27^ Nevertheless, gelatin hydrogels alone lack the mechanical strength for these applications, especially at temperatures above 29 °C.^28^ Consequently, several strategies have been explored to overcome this challenge, including functionalization with methacrylate groups^29^ for ultraviolet (UV) cross-linking (as is the case of the several commercial inks, e.g., PhotoGel-INK,^30^ GelMA PhotoInk,^31^ GelMA Bioink,^32^ and GelXG^33^ from CELLINK). UV-based cross-linking has been widely used and reported in many studies;^34^ however, the need for the chemical modification of the biopolymer, the use of photoinitiators, and the potential impact of this strategy in cell viability, for example, as a result of DNA damage may limit the application of this methodology.^35^ In this context, combining gelatin with natural-based nanofibers, for instance nanocellulose, represents a safer strategy.^36,37^ Nanofibrillated cellulose (NFC) is obtained through the disintegration of cellulose fibers (e.g., from wood, cotton, and bamboo) by combining intense mechanical treatments and chemical or enzymatic approaches.^38^ This treatment translates into long, flexible, and entangled nanosized cellulose fibrils, with diameters in the range of 5–60 nm and several micrometers in length, and with alternating crystalline and amorphous domains.^38,39^ NFC and gelatin have been combined in previous studies to create bioinks for 3D bioprinting;^40–44^ however, the reported approaches still required chemical modification (of gelatin or nanocellulose) or the addition of other polymers to fabricate hydrogel bioinks with adequate performance. Specifically, these studies include blending NFC and gelatin methacrylate (GelMA) for UV cross-linking,^40,41^ carboxymethylation of NFC and its mixing with gelatin for cross-linking with microbial transglutaminase,^42^ and the combination of NFC and gelatin with a third biopolymer, namely, alginate, for the subsequent cross-linking with CaCl_2_.^43^

In this study, we propose the development of Gel/NFC hydrogel-based bioinks for the 3D bioprinting of structures with improved features, using genipin as gelatin cross-linking agent, eliminating the need for additional chemical modifications or the use of complex polymeric mixtures. Genipin is an aglycone derivative, obtained by the hydrolysis of geniposide, which is extracted from the fruits of *Gardenia jasminoides*, and commonly used to cross-link biopolymers with amino groups, as proteins or polysaccharides like chitosan.^45,46^

Herein, Gel/NFC inks with different compositions were prepared and characterized considering their rheological properties, and the corresponding cross-linked hydrogels were then evaluated in terms of their rheological and mechanical performance, degradability, morphology, and cytotoxicity toward the human hepatoma cell line (HepG2). The hydrogel-based ink with the most promising properties was finally loaded with HepG2 cells, and the cell viability was assessed on days 1, 3, 7, and 14 after 3D bioprinting. Moreover, the culture media of the 3D bioprinted cells at day 7 postbioprinting were analyzed by proton nuclear magnetic resonance (^1^H NMR) spectroscopy to better understand the impact of the bioprinting process on the cell’s metabolism and the ability of the developed bioink to create an adequate environment for cells to thrive.

## 2 Materials and Methods

### 2.1 Materials

The NFC suspension (2% wt.) was produced from softwood bisulfite fibers using a combination of mechanical and enzymatic processes to produce fibrils with an average diameter of 20–50 nm and a carboxyl content of 0.14–0.06 mmol g^–1^,^47^ kindly supplied by VTT Technical Research Centre (Finland). Sodium pyruvate (100 mM) was provided by Biochrom GmbH (Berlin, Germany). 3-(4,5-Dimethylthiazolyl-2)-2,5-diphenyltetrazolium bromide (MTT, 98%) was supplied by Alfa Aesar (Kandel, Germany). Gelatin from porcine skin (gel strength 300, type A), dimethyl sulfoxide (DMSO, ≥99.5%), phosphate buffer saline (PBS, pH 7.4), 3-(trimethylsilyl) propionic acid (TSP-*d*_4_) 0.75% in deuterium oxide (D_2_O), and D_2_O 99.9 atom % D were supplied by Sigma-Aldrich (Sintra, Portugal). Dulbecco’s Modified Eagle’s Medium (DMEM) and fetal bovine serum (FBS) were purchased from PAN-Biotech (Aidenbach, Germany). l-Glutamine and penicillin/streptomycin were obtained from GRiSP (Porto, Portugal). Fungizone was supplied by Gibco (Life Technologies, Carlsbad, CA, USA). LIVE/DEAD cell viability assay kit was supplied by Sigma-Aldrich (Sintra, Portugal). Ultrapure water (type 1, 18.2 MΩ cm resistivity at 25 °C and 0.5 L min^–1^) was obtained with a Simplicity Water Purification System (Merck, Darmstadt, Germany). Genipin was obtained from Challenge Bioproducts Co. (Taiwan, China).

The HepG2 cell line, a hepatocellular carcinoma cell line, was acquired from the European Collection of Authenticated Cell Cultures (ECACC) and supplied by Sigma-Aldrich (St. Louis, MO).

### 2.2 Preparation of the Gel/NFC-Based Inks and Cross-Linked Hydrogels

The excess supernatant of the NFC suspension was previously removed by centrifugation (6000 rpm for 20 min) to increase the solid content of NFC from 2 to 4% (w/v). Next, adequate quantities of Gel and ultrapure water were added into the NFC suspension to obtain four Gel/NFC formulations with different mass fractions, namely, 90:10, 80:20, 70:30, and 60:40, all in a final volume of 10.0 mL. The final concentration of each component in the inks is listed in Table 1. Each formulation was intensively mixed to obtain homogeneous ink suspensions. Genipin was finally added to the different Gel/NFC inks at a concentration of 1.5 wt %, referring to the mass of the Gel. The formulations were incubated at 37 °C overnight to promote the cross-linking reaction, resulting in cross-linked hydrogels used for characterization purposes.

**Table 1:** Summary of the Different Gel/NFC Inks

Gel/NFC (w/w)	Gel (w/v)	NFC (w/v)
90:10	9	1
80:20	8	2
70:30	7	3
60:40	6	4

### 2.3 Physicochemical Characterization of the Gel/NFC Inks and Corresponding Cross-Linked Hydrogels

#### 2.3.1 Rheological Characterization

The rheological evaluation of the developed Gel/NFC inks was carried out using a Kinexus Pro (Malvern Instruments, Malvern, U.K.) equipped with parallel-plate geometry. Each ink formulation was positioned in the measuring gap and left to equilibrate at the defined temperature for 5 min. The data obtained from these characterizations were presented as the mean of three replicates and the corresponding standard deviation.

A temperature sweeping test was carried out to determine the temperature at which the sol–gel transition occurs. Each ink underwent a stage of cooling from 40 to 20 °C at a rate of 5 °C min^–1^ and a 1 mm gap. Then, all inks were subjected to a heating stage from 20 to 40 °C, also at a rate of 5 °C min^–1^. Both gelling and melting temperatures were defined as the temperature where the crossover of storage (*G*′) and loss (*G*″) moduli occurred or where an inflection was observed (in the cases where a crossover did not occur).

The shear viscosity of the inks was acquired in a shear rate range between 0.1 and 100 s^–1^ at 30 °C, with a 1 mm gap. The data obtained were adjusted using the power-law model^48^ defined as eq 1

$$\eta =K\gamma^{\left(n-1\right)}$$ 1

where η is the shear viscosity (Pa·s), *K* is the consistency index (Pa·s), γ is the shear rate (s^–1^), and *n* is the flow index.

The yield stress of the hydrogel-based inks was determined through an oscillatory shear stress sweep test in the linear viscoelastic region at 1 Hz and in a shear stress range from 1 to 700 Pa and 1 mm gap. The shear stress at the crossover point between the *G*′ and *G*″ moduli is defined as the yield stress of the corresponding ink.

The corresponding cross-linked hydrogels were assembled into disc specimens with an average diameter of 1.5 cm and a thickness of 5.0 mm. The obtained specimens were subjected to an oscillatory strain sweep test, conducted in a strain range between 0.1% and 100%, at a frequency of 1 Hz, 37 °C, and a 5 mm gap. The extent of the gel-like behavior of the hydrogels was studied through the calculation of the tangent of the phase angle, also known as loss tangent, using eq 2

2 $$\tan\delta =G^{\prime \prime }/G^{\prime }$$

where δ is the phase angle.

#### 2.3.2 Gel Fraction Determination

The gel fraction of the cross-linked hydrogels was determined using disc specimens after gelation.^49^ The samples were first oven-dried at 60 °C for 72 h to determine their initial dried weight (Wdi). Then, they were immersed in ultrapure water for 24 h, at 60 °C, to promote the removal of the non-cross-linked polymeric fraction. Afterward, the washed samples were once again dried under the same conditions and reweighed to obtain the final dried weight (Wdf). The gel fraction percentage was calculated based on eq 3

3 gelfraction(%)=Wdf/Wdi×100

#### 2.3.3 Mechanical Properties

Compression tests of the resulting cross-linked Gel/NFC hydrogels in the wet state were carried out using a uniaxial Instron 5966 testing apparatus (Instron Corporation, Norwood, MA, USA) equipped with a 500 N load cell, at room temperature and using specimens with about 1.5 cm diameter and 5.0 mm of thickness. The measurements were conducted in unconfined compression with a strain rate of 10%·s^–1^ until reaching a compressive strain of 80%. Similar tests were also carried out for hydrogel samples at different incubation time points (up to 14 days) in DMEM at 37 °C. The compressive stress and Young’s modulus were calculated using Bluehill 3 Software (Version 3.22, Illinois Tool Works Inc., Glenview, IL, USA). The obtained results were provided as the mean of five replicates and the corresponding standard deviation.

#### 2.3.4 Degradation Assay

To evaluate the degradation rate of the cross-linked Gel/NFC hydrogels in PBS and DMEM, the weight loss over time was monitored, up to 72 h and 14 days, respectively. For that, the samples were previously immersed overnight in ultrapure water to achieve a swollen equilibrium state. Then, they were weighed and immersed in PBS or DMEM at 37 °C. The samples were removed from the respective media and weighed at specific time points. Measurements were carried out using three replicates of each Gel/NFC hydrogel, and the results are presented as the mean and the respective standard deviation. The stability of the hydrogels was assessed by calculating the degradation rate with eq 4

4 $$degradation\;\left(\%\right)=\left(w_{i}-w_{t}\right)/w_{i}\times 100$$

where *w*_*i*_ is the initial weight of each sample and *w*_*t*_ is the weight of each sample at each time point.

### 2.4 3D Printing

The printing assays were performed using an extrusion-based 3D-Bioplotter System from Desktop Health (EnvisionTEC GMBH, Gladbeck, Germany). The printing parameters, viz, nozzle inner diameter, printing pressure, and speed, were previously adjusted. After this optimization step, grid-like models with 2 layers (20 × 20 mm^2^, a single layer height of 0.320 mm, 1.6 mm of filament distance, and a printing angle of 90°) were printed. Extrusion printing was carried out at 30 °C, with the printing platform at room temperature. Printing experiments were performed for all of the formulations listed in Table 1, with genipin being added immediately before the extrusion procedure. The printed structures were incubated at 37 °C, overnight, to promote the genipin-mediated cross-linking.

#### 2.4.1 Printability

The printability index (Pr) of the 2-layered Gel/NFC-grid models was calculated with eq 5

5 $$\mathrm{Pr}=L^{2}/16A$$

where *L* is the perimeter, and *A* is the area of the pores of the 2-layered printed constructs.

Furthermore, the printing fidelity/quality (φ) was evaluated using eq 6, designed by He et al.^50^

6 $$\varphi =\left(A_{rt}-A_{re}\right)/A_{rt}$$

where *A*_rt_ is the theoretical area of the lattice and *A*_re_ is the experimental one.

#### 2.4.2 Morphology of the 3D Printed Constructs

The morphology of the freeze-dried 3D printed constructs was evaluated by scanning electron microscopy (SEM) and the micrographs recorded in an HR-FESEM SU-70 Hitachi microscope (Hitachi High-Technologies Corporation, Tokyo, Japan) operated at 4.0 kV. The samples were placed on an aluminum plate and carbon coated using an EMITECH K950 coating system.

#### 2.4.3 Porosity Degree

Freeze-dried cross-linked Gel/NFC printed samples were used for the porosity assessment through a liquid displacement method, following a methodology similar to the one reported by Moshayedi et al.^51^ Ethanol was chosen as the displacement liquid because of its capacity to infiltrate the pores of the Gel-based hydrogels without inducing shrinking or swelling.^52,53^ The weight and volume of the freeze-dried hydrogels (three replicates) were measured before being immersed in ethanol for 24 h. Data were presented as the mean of the replicates and the corresponding standard deviation. After the stipulated time, the excess ethanol was removed and the hydrogels were weighed. Porosity was calculated using the following equation

7 $$porosity\;\left(\%\right)=\left(w_{f}-w_{i}\right)/\left(\rho \times V\right)\times 100$$

where *w*_f_ is the final weight of the hydrogels, *w*_*i*_ is the initial weight, ρ is the density of ethanol at room temperature (0.785 g cm^–3^), and *V* is the volume of the freeze-dried hydrogel samples.

### 2.5 In Vitro Cytotoxicity of the Gel/NFC Hydrogels toward HepG2 Cells

#### 2.5.1 Cell Culture

HepG2 cells were seeded and cultured in a T25 flask (25 cm^2^) in DMEM supplemented with 10% FBS, 100 U mL^–1^ penicillin, 10 μg mL^–1^ streptomycin, 2 mM l-glutamine, 250 μg mL^–1^ fungizone, and 1% sodium pyruvate, at 37 °C in a humidified atmosphere with 5% CO_2_. Cells were examined on a regular basis under an inverted phase-contrast Eclipse TS100 microscope (Nikon, Tokyo, Japan), and when the confluence reached 80–90%, adherent cells were detached using 0.25% trypsin. The obtained cell suspension in DMEM was used for the following assays.

#### 2.5.2 In Vitro Cytotoxicity

The cytotoxicity of the Gel/NFC hydrogels was assessed toward the HepG2 cell line. Before the hydrogels were prepared, each component was sterilized. Specifically, NFC and ultrapure water were autoclaved (100 kPa, 121 °C for 20 min), and Gel and genipin powders were sterilized under UV light (3 cycles, 20 min each). The hydrogel extracts (25 mg mL^–1^) were prepared according to the methodology described in Section 2.2, under sterile conditions, and incubated for 24 h in DMEM.

Cells were seeded (150,000 cells mL^–1^ for 24 h and 100,000 cells mL^–1^ for 48 h) in 96-well plates and incubated in DMEM to promote cell adhesion. Three independent assays and six replicates for each condition were carried out. After 24 h, the culture medium was substituted by 100 μL of the hydrogel’s extract, and cells were further incubated for 24 and 48 h. HepG2 cells were also incubated with DMEM and treated in the same way as the specimens to act as the control. After 24 and 48 h of incubation, 50 μL of MTT (1 mg mL^–1^) in PBS was added to each wall and incubated for 4 h at 37 °C with 5% CO_2_. Then, the medium containing MTT was removed and replaced with 150 μL of DMSO. To enable the dissolution of the produced formazan crystals, the plate was shaken in the dark for 2 h. Using a BioTek Synergy HT plate reader (Synergy HT Multi-Mode, BioTek, Winooski, VT), the absorbance was acquired, and the cell viability was calculated according to eq 8

8 $$cellviability\;\left(\%\right)=\left[\left(Abs_{sample}-Abs_{DMSO}\right)/\left(Abs_{control}-Abs_{DMSO}\right)\right]\times 100$$

where Abs_sample_ is the absorbance of the sample, Abs_DMSO_ is the absorbance of DMSO, and Abs_control_ is the absorbance of the control.

### 2.6 3D Bioprinting of HepG2-Laden Hydrogels

The ink formulation with a Gel/NFC composition of 70:30 was selected for the incorporation of cells. The ink was prepared as described in Section 2.2, under sterile conditions (as referred to in Section 2.5.2). The cell pellet, obtained following a centrifugation step for 30 min at 6000 rpm, was resuspended in 1 mL of DMEM and gently mixed with the ink at a final concentration of 2 × 10^6^ cells mL^–1^. All accessories and the bioprinter were sanitized using 70% ethanol before the bioprinting procedure. HepG2-ladden grids of 20 × 20 mm, 1.6 mm of filament distance, and 2 layers were bioprinted using a 0.41 mm nozzle (inner diameter), with a printing pressure of 2 bar and a printing speed of 10 mm s^–1^. After bioprinting, the structures were submerged in DMEM and incubated for up to 14 days. On days 1, 3, 7, and 14 after bioprinting, the culture media were removed, and the structures were washed with sterile PBS. At those time points, PBS containing the LIVE/DEAD reagents (propidium iodide/calcein AM) was added to each Petri dish. The cell-laden 3D bioprinted structures were observed using a confocal microscope (Zeiss LCM 770, Carl Zeiss, Oberkochen, Germany), obtaining a *Z*-stack image of 15 layers across the whole height of the structures on, at least, 3 different regions of each 3D structure, where live cells were labeled in green and the dead cells in red. Three replicates were performed. Cell viability was calculated using eq 9

9 $$cellviability\;\left(\%\right)=\frac{greenintensity}{\left(red+greenintensity\right)}\times 100$$

### 2.7 Metabolic Evaluation of HepG2 Cells

#### 2.7.1 Cell Treatment and Sample Collection for ^1^H NMR Analysis.

Bioprinted HepG2-laden hydrogels were incubated in culture medium at 37 °C under a humidified atmosphere with 5% CO_2_. 3D-printed structures (without cells) were also incubated under the same conditions to act as controls. Three replicates were carried out for each condition.

After 7 days, the medium of each sample was collected and centrifuged at 1000*g* for 5 min at 4 °C. Subsequently, aliquots of 350 μL were frozen at −80 °C. To precipitate the medium proteins and preserve the metabolites, 700 μL of cold methanol (prestored at −80 °C) was added to a medium aliquot and placed at −20 °C for 30 min. Then, samples were centrifuged at 13,000*g* for 20 min, and the supernatant was collected, dried in a CentriVap (model 7300, Labconco, Kansas City, MO), and stored at −80 °C.

At the time of NMR analysis, 550 μL of deuterated PBS (pH 7.4, 100 mM, containing 0.1 mM TSP-*d*_4_) was added to each sample and transferred into 5 mm NMR tubes.

#### 2.7.2 NMR Data Acquisition

Standard 1D ^1^H NMR spectra were acquired on a Bruker Avance HD III 500 spectrometer (University of Aveiro, Portuguese NMR Network), operating at 500.13 MHz for ^1^H observation, at 298 K. The pulse program “noesypr1d” was employed, with the following main parameters: 7002.8 Hz spectral width, 32 k data points, 2 s relaxation delay, and 512 scans. Topspin 4.0.6 (Bruker BioSpin, Rheinstetten, Germany) was used for spectral processing, which included manual phasing, baseline correction, and signal calibration to the TSP-*d*_4_ signal (0 ppm). For quantitative measurement of metabolic variations, the selected signals, representative of individual metabolites, were integrated with Amix-Viewer 3.9.15 (Bruker Biospin). Signal areas were then used to calculate the percentage of variation of each metabolite in cell-conditioned vs acellular medium. The variations of higher magnitude (|effect size| > 1.2) were represented graphically using Origin Pro 2018 (OriginLab Corporation, Northampton, MA, USA).

### 2.8 Statistical Analysis

The statistical analysis was carried out using a one-way analysis of variance (ANOVA) followed by a Tukey’s test using Origin Pro 2018, with a statistical significance determined at 95% confidence level.

## 3 Results and Discussion

This work aimed at developing composite hydrogel-based bioinks composed of NFC and Gel, by the simple cross-linking with genipin, for the 3D bioprinting of HepG2 cells (Figure 1). NFC was selected due to its superior rheological and mechanical properties, while Gel was chosen since it retains the tripeptide RGD sequence of collagen, which promotes cell adhesion and proliferation. The cross-linking of gelatin, which was essential for the final properties of the 3D structures, was mediated by genipin, avoiding the need for additional chemical modifications of the biopolymers. This cross-linking is based on the nucleophilic attack of the amino group of Gel to the olefinic carbon atom at C3 of genipin, leading to the formation of an amide bond.^46^ Gel/NFC inks with different mass compositions, viz, 90:10, 80:20, 70:30, and 60:40, were prepared with the aim of attaining adequate properties for the 3D bioprinting of liver cancer cells. All Gel/NFC mixtures had a paste-like consistency, and the corresponding cross-linked hydrogels had a blueish appearance, as observed in the printed structure represented in Figure 1, due to their cross-linking with genipin. The developed inks were characterized concerning their rheological properties (shear-thinning, yield stress, and temperature sweeping test), and the resulting cross-linked hydrogels were evaluated regarding their rheological and mechanical properties, stability in DMEM and PBS, printability, morphology, and cytotoxicity toward HepG2 cells. The viability of the cells laden on the 3D bioprinted structures was assessed using a LIVE/DEAD test on days 1, 3, 7, and 14 following 3D bioprinting. The cells’ metabolic activity was also studied on day 7 after bioprinting in order to assess whether the HepG2 cells’ metabolism is maintained in the 3D bioprinted constructs.

**Figure 1:**
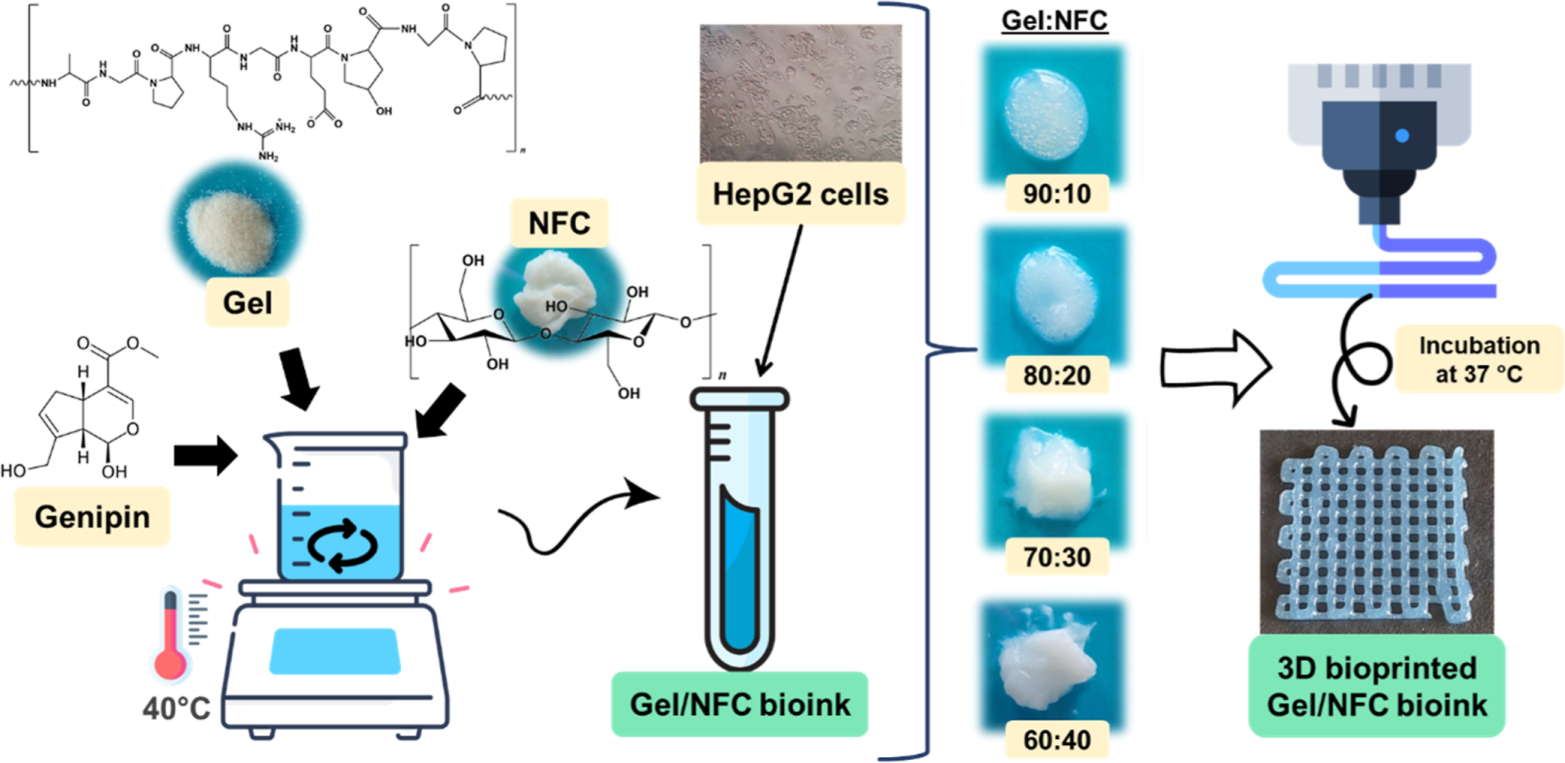
Schematic representation of the main steps involved in the preparation of the Gel/NFC bioinks for 3D bioprinting of liver cancer cells.

### 3.1 Physicochemical Characterization of the Gel/NFC Inks and the Corresponding Cross-Linked Hydrogels

#### 3.1.1 Rheological Characterization

The rheological properties of the hydrogel-based bioinks strongly dictate their printability and, therefore, the potential for bioprinting endeavors.^54^ Thereby, the rheological properties of the developed Gel/NFC hydrogel-based inks were evaluated to fully grasp their performance during the 3D printing process.

An ideal hydrogel bioink should have a shear-thinning behavior, that is, the shear viscosity should decrease with increasing shear rate to facilitate its extrusion through the nozzle.^19^ Since gelatin-based inks are thermoresponsive, the best temperature for printing must be previously determined to avoid nozzle clogging due to its thermal cross-linking. Thus, each hydrogel-based ink underwent a temperature sweeping test that included a cooling stage from 40 to 20 °C to determine its gelling temperature and a heating stage from 20 to 40 °C to determine the melting temperature. From the results obtained, it is possible to notice (Figure 2A) that the values of *G*′ and *G*″ increased as the temperature dropped, reaching an inflection point starting at about 25 °C. For the 90:10 ink, a crossover between *G*′ and *G*″ was visible at 22 °C, which is the exact temperature at which the sol–gel transition took place. Higher NFC contents (80:20, 70:30, and 60:40) translated into the disappearance of this crossover and a less prominent inflection. Likewise, Liu and co-workers^55^ observed that increasing the concentration of nanoattapulgite on gelatin methacrylate-based bioinks translated into the disappearance of the crossover point between *G*′ and *G’‘*. In the heating stage, represented in Figure 2B, a deflection point was observed at about 30 °C for all inks. However, similarly to the cooling stage, this point became less prominent as the NFC content increased. These findings demonstrated the key role of NFC in improving the stability of the Gel/NFC inks throughout the tested temperatures and revealed that the optimal printing temperature range should be between 25–32 °C.

**Figure 2:**
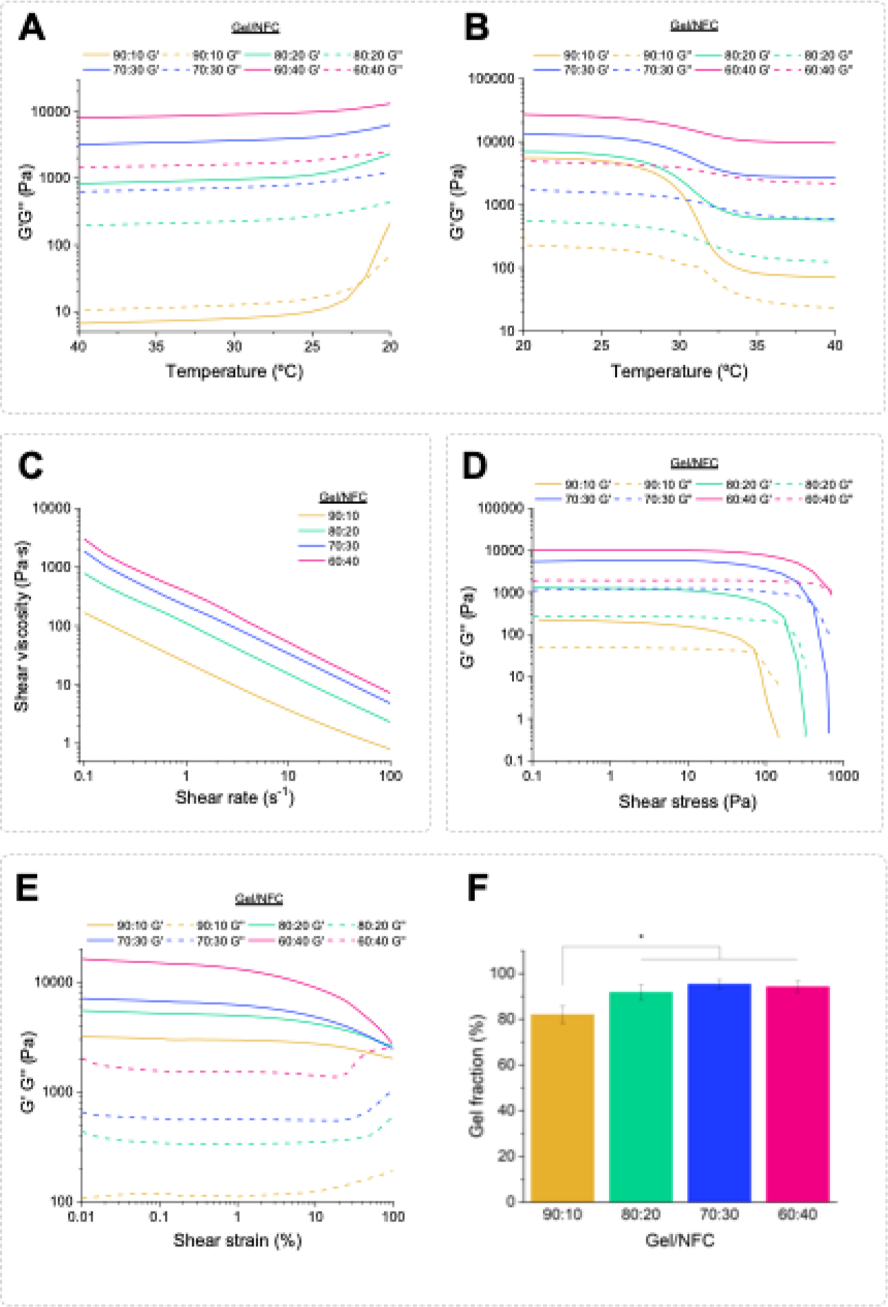
*G*′ and *G*″ moduli of the Gel/NFC inks over a (A) cooling step and (B) heating step; (C) flow curves of the different Gel/NFC inks; (D) yield stress as a function of shear stress of the formulated inks; (E) plot of *G*′ and *G*″ moduli as a function of the shear strain of the corresponding cross-linked hydrogels; and (F) gel fraction of the formulated Gel/NFC hydrogels. Results are shown as the mean of 5 replicates, and the error bars are the standard deviation (*p* < 0.05: *).

Considering these results, the viscosity of all formulated Gel/NFC inks was evaluated at 30 °C. As highlighted in Figure 2C, all formulations exhibited a clear shear-thinning behavior, observed by the decrease in shear viscosity with the shear rate. Furthermore, the extent of the shear-thinning behavior of the formulated inks was adjusted to the power-law model (eq 1).^48^ The fluid index (*n*) is a parameter that elucidates the fluid behavior of a material and should be lower than 1 for non-Newtonian fluids. All the prepared Gel/NFC formulations showed a proper shear-thinning behavior with decreasing *n* values as the NFC content increased, specifically 0.155 ± 0.002, 0.120 ± 0.017, 0.081 ± 0.019, and 0.076 ± 0.026 for inks 90:10, 80:20, 70:30, and 60:40, respectively. Similar tendencies were reported by Tuladhar et al.^56^ and Lan et al.^57^ for increasing amounts of NFC on carboxymethyl cellulose and alginate hydrogel-based inks, respectively. Another important parameter to take into consideration is the consistency index (*K*), which is directly proportional to the shear viscosity of the material. It was also noticed that increasing the content of NFC in the inks translated into an increase in *K* values, viz 23.8 ± 0.1, 100.8 ± 3.5, 182.2 ± 11.6, and 297.8 ± 23.5 for inks 90:10, 80:20, 70:30, and 60:40, correspondingly. Again, Lan et al.^57^ described an analogous behavior for alginate/NFC hydrogel inks. Additionally, the initial shear viscosity (measured at a shear rate of 0.1 s^–1^) of the four formulations increased with the amount of NFC, specifically from 161 ± 88 Pa·s for the hydrogel 90:10 to 1664 ± 109 Pa·s for the hydrogel 60:40.

The yield stress is a parameter that expresses the minimum stress necessary for a bioink to start flowing through the nozzle.^58,59^ This parameter was determined as the crossover point between *G*′ and *G*″ of the plotted curves as a function of the shear stress (Figure 2D). Yield stress increased with increasing contents of NFC, with inks 90:10, 80:20, 70:30, and 60:40 showing values of 70.9 ± 28.6, 219.3 ± 6.9, 404.0 ± 13.6, and 627.9 ± 74.8 Pa, respectively. These results indicate that increasing contents of NFC strengthen the inks, expressed by increasing their resistance to the shear stress applied. Radeke et al.^42^ and Shin et al.^41^ also reported an increase in the yield stress for Gel/carboxymethylated NFC (cNFC) and GelMA/NFC hydrogel-based formulations with increasing contents of cNFC or NFC, respectively.

For the cross-linked hydrogels, the results of both *G*′ and *G*″ over a shear strain range of 0–100% are shown in Figure 2E. All samples disclosed *G*′ values higher than *G*″ over the studied shear strain range, indicating their gel-like nature and therefore confirming the success of the cross-linking with genipin.^60^ Additionally, both *G*′ and *G*″ values of the cross-linked hydrogels also increased with the rising amount of NFC, confirming its reinforcing effect, as previously observed for the Gel/NFC ink formulations. A comparable behavior was observed by Luo et al.^43^ when adding NFC to Gel-alginate-based inks. To further evaluate the gel-like behavior of the cross-linked hydrogels, the calculation of the loss tangent (tan δ, eq 2) in the linear viscoelastic region was performed. The tan δ values were lower than 1 for all cross-linked hydrogels, which proved their gel-like behavior.^60^ Moreover, the gel fraction of these samples was also determined to infer on the cross-linking extent for each formulation (Figure 2F). The calculated percentages varied between 82.1 ± 3.8% (90:10), 91.6 ± 3.3% (80:20), 95.5 ± 2.0% (70:30), and 94.3 ± 2.5% (60:40). All values are close to 100%, which indicates the nearly total cross-linking of hydrogels.

#### 3.1.2 Mechanical Properties

In order to investigate if the bioinks are able to maintain their shape after printing and act as a mechanical support for cells, cross-linked hydrogels were studied for their mechanical properties.^61^ The results of the compressive assays of the different hydrogels are presented in Figure 3A. The hydrogels with the lowest content of NFC, viz, 90:10 and 80:20, showed the lowest Young’s modulus with values of 4.7 ± 0.9 MPa and 5.1 ± 0.9 MPa, respectively. The samples with higher contents of NFC reached values of 13.6 ± 2.1 MPa and 12.1 ± 1.1 MPa, specifically for the hydrogels 70:30 and 60:40, correspondingly. A similar trend was observed for compressive stress at 80% strain, with hydrogels 90:10 and 80:20 showing the lowest values (0.5 ± 0.1 and 0.6 ± 0.1 MPa, respectively) and higher values for hydrogels 70:30 and 60:40 (1.7 ± 0.4 and 1.5 ± 0.2 MPa, in that order), as can be seen in Figure 3B. These obtained results are in accordance with the rheological analysis, confirming the importance of NFC to fabricate Gel-based bioinks with superior rheological and mechanical properties for bioprinting demands.

**Figure 3:**
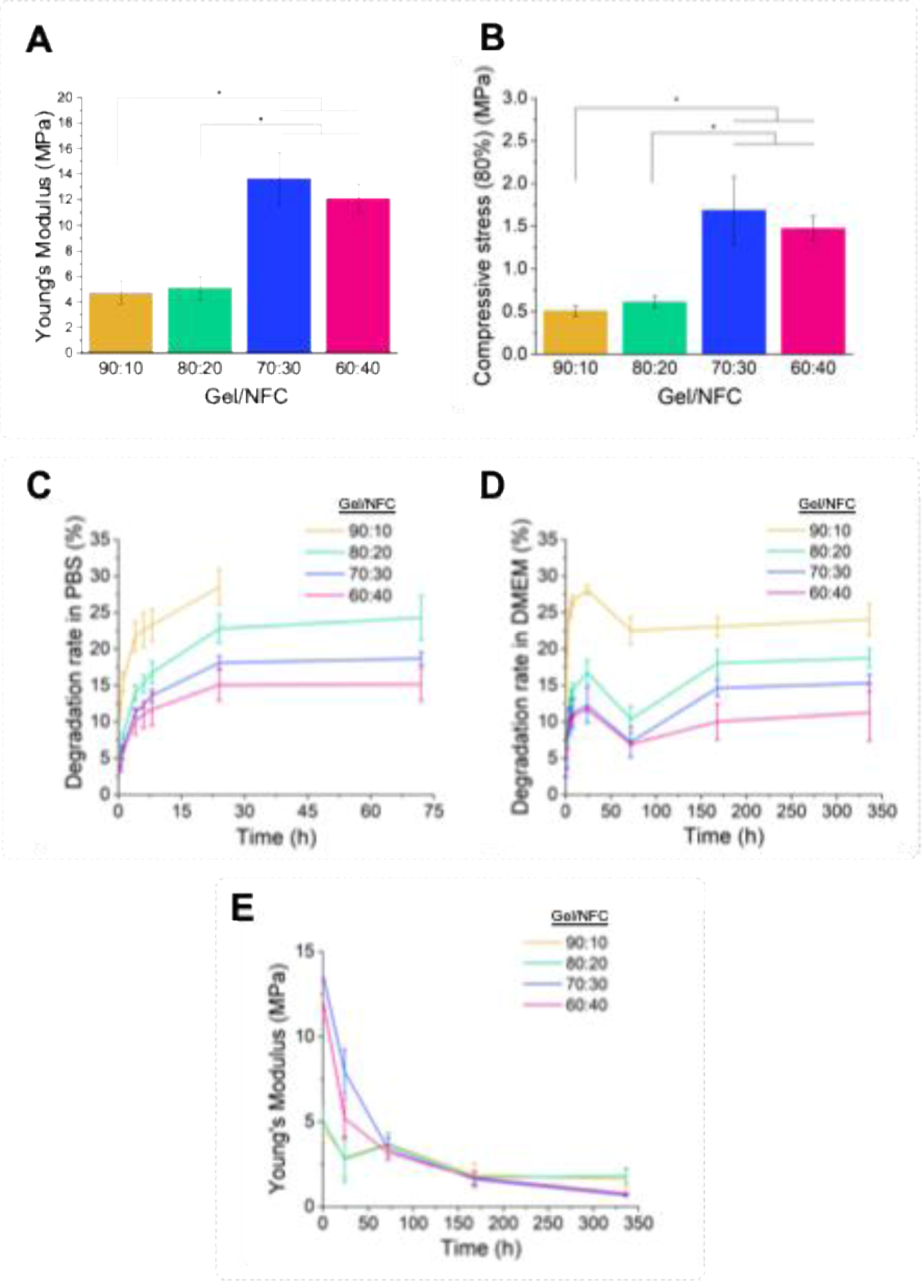
(A) Young’s modulus and (B) compressive stress at 80% strain of the cross-linked Gel/NFC hydrogels. Results are shown as the mean of 5 replicates, and the error bars are the standard deviation (*p* < 0.05:*). Degradation profiles of the Gel/NFC hydrogels in (C) PBS (up to 72 h) and (D) DMEM (up to 14 days) at 37 °C. (E) Evolution of the Young’s modulus of the cross-linked Gel/NFC hydrogels after the incubation in DMEM at 37 °C in different time points (0, 1, 3, 7, and 14 days). Data are presented as the mean of 3 replicates, and the bars represent the standard deviation.

#### 3.1.3 Degradation Assay

The degradation of the cross-linked hydrogels was studied by monitoring the mass loss at selected time points over 3 days, in two different media, one simulating the physiological conditions (PBS, pH 7.4) and the other the incubation conditions of the 3D bioprinted constructs in culture media (DMEM) at 37 °C. For both media (PBS and DMEM), the degradation rate (Figure 3C,D) diminishes with increasing NFC content because of its resistance to degradation in different media due to its high crystallinity and fibrillar structure.^62^ All hydrogels showed rapid degradation in the first 8 h, reaching a maximum after 24 h. At this point, the weight losses were very similar between the two media. Specifically, in PBS, weight losses of 28.5 ± 2.5%, 22.8 ± 2.0%, 18.1 ± 0.9%, and 15.1 ± 2.2% were seen for inks 90:10, 80:20, 70:30, and 60:40, respectively. In DMEM, the weight losses observed were 28.1 ± 0.7%, 16.8 ± 1.7%, 12.4 ± 2.5%, and 11.7 ± 0.2% for inks 90:10, 80:20, 70:30, and 60:40, respectively. In PBS, the hydrogel 90:10 disintegrates completely after 24 h of incubation (Figure 3C), which might be related to the dissolution of gelatin due to the disruption of the genipin-mediated cross-linking.^63^ At 72 h of incubation in DMEM, a slight decrease in weight loss was observed that could be associated with the absorption of the culture medium. Interestingly, this only happened in DMEM, which might be related to the complexity of the culture medium, compared to PBS. In addition, to further investigate the degradation behavior of the cross-linked hydrogels in cell culture conditions, the assay in DMEM was extended for up to 14 days (Figure 3D). At this point, the degradation rates did not surpass the maximum values reached at 24 h, confirming the stability of the hydrogels within this time frame.

#### 3.1.4 Effect of Incubation in DMEM in the Mechanical Properties of the Hydrogels

Nonetheless, the gradual degradation of the hydrogels observed over a period of 14 days may affect their mechanical behavior. Therefore, in order to explore the impact of this phenomenon, the stiffness of the hydrogels was evaluated during this time period of incubation in DMEM. The results depicted in Figure 3E show that all the cross-linked hydrogels presented a gradual decrease on their Young’s modulus, consistent with the degradation assays described before. In fact, the final Young’s modulus observed for 70:30 and 60:40 samples decreased up to 18-fold, reaching around 700 kPa, which is nearer to the range of fibrotic hepatic tissue.^64^ The superior mechanical properties of the Gel/NFC hydrogels allow the bioprinting of stable 3D structures that, when incubated in DMEM medium at 37 °C, reaches adequate stiffnesses that guarantee cell survival and proliferation, as will be discussed below.

### 3.2 3D Printing

The evaluation of the printability of the developed Gel/NFC formulations was posteriorly carried out to confirm their potential application for 3D bioprinting endeavors. The first step of this procedure consisted of the optimization of the printing parameters, namely, nozzle inner diameter, printing speed, and pressure, by printing straight filaments of 750 mm length and evaluating filament breaking and spreading. The results showed that the inks could be successfully printed with a nozzle inner diameter of 0.41 mm, a printing speed of 10 mm s^–1^, and pressure ranging between 1 and 2 bar, depending on the Gel/NFC mass composition (1 bar for the inks 90:10 and 80:20, and 2 bar for the 70:30 and 60:40 ones). Afterward, grid-like models with 20 × 20 mm, a single layer height of 0.320 mm, a filament distance of 1.6 mm, and 2 layers were printed using the optimized printing parameters and all ink formulations. A color change was perceived in all these 3D printed constructs, resulting from the cross-linking with genipin. Additionally, the results obtained showed that the increasing amount of NFC yielded 3D constructs with better shape fidelity and resolution (Figure 4A), which is in accordance with the rheological and mechanical data previously discussed. This translates into structures that are more stable and easier to handle (Figure 4B). The printability index (Pr), a parameter that evaluates the printing quality of the 3D constructs, was calculated for each structure. To be adequate for extrusion bioprinting, a bioink must have a printability index within the range of 0.9–1.^54^ Between the hydrogels studied here, only those with higher NFC contents met this requirement, namely, the hydrogel inks 80:20 (0.90), 70:30 (0.92), and 60:40 (0.94).^54,60^ Printing fidelity was further evaluated using eq 5, defined by He et al.,^50^ and this parameter refers to the ability of the printed structure to maintain its shape after extrusion. According to the equation, a suitable bioink for 3D printing must have a printing fidelity lower than 1 and as close to 0 as possible.^60^ All developed hydrogels showed values lower than 1, viz, 0.918 ± 0.002, 0.798 ± 0.007, 0.622 ± 0.009, and 0.307 ± 0.021, for 90:10, 80:20, 70:30, and 60:40, respectively, and were statistically different, confirming that the formulations with higher NFC content have better performance.^60^ In fact, these results of printability and printing fidelity are coherent and in accordance with the rheological properties of the formulations, where the inks with higher NFC content showed higher shear viscosity while maintaining a clear shear thinning behavior.

**Figure 4:**
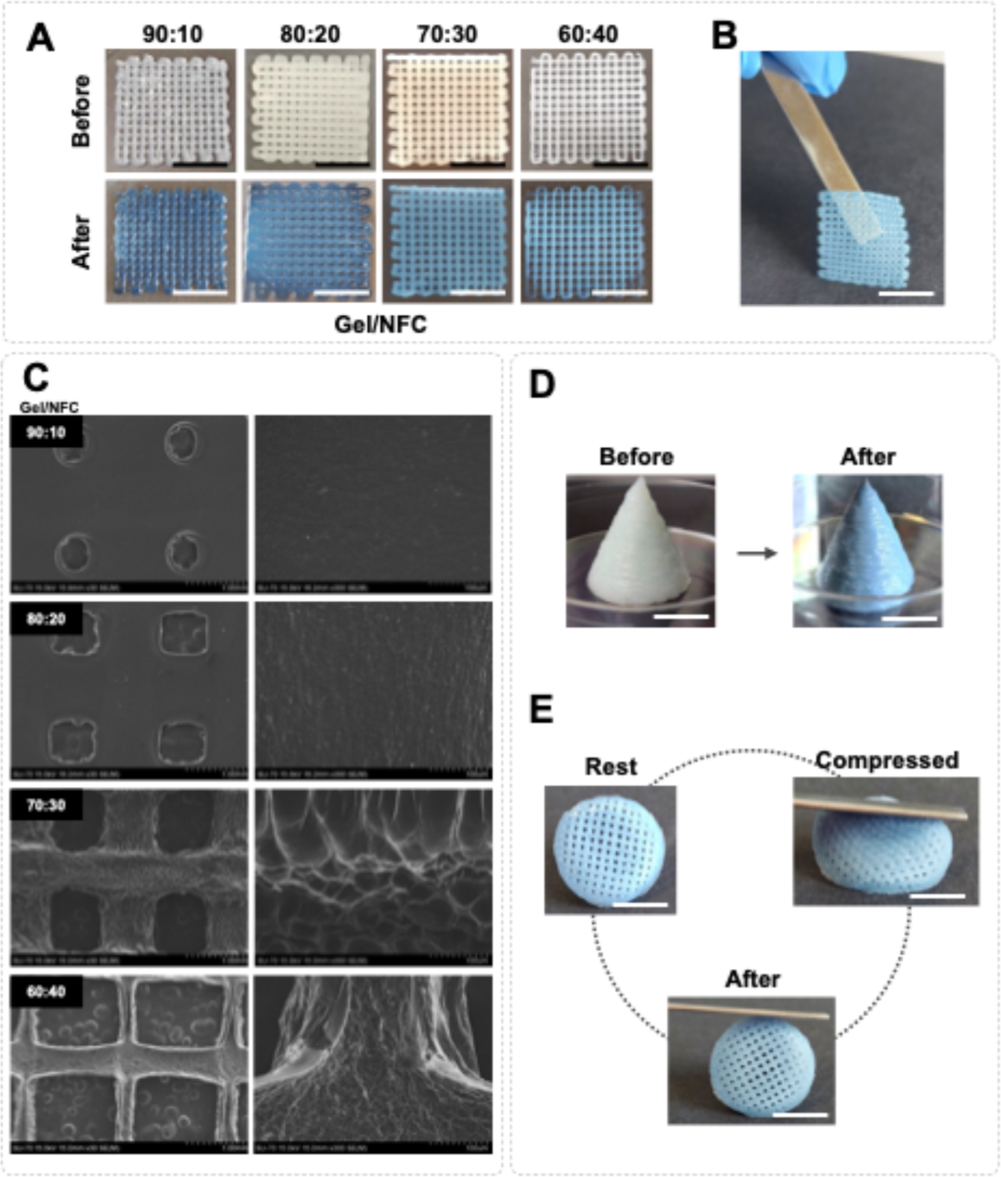
(A) Images of the Gel/NFC-grid-like 3D constructs before and after cross-linking with genipin; (B) image of the cross-linked 70:30 hydrogel evidencing the ease of handling the structure; (C) SEM surface micrographs of the freeze-dried 3D printed structures (magnification is ×30 (left) and × 300 (right), respectively); (D) images of a 3D printed cone (70:30 ink), before and after cross-linking; and (E) image of a 3D printed structure (70:30 ink) before, during, and after manual compression with a spatula. Scale bars = 10 mm.

The morphological characteristics of the cross-linked 3D printed structures, assessed by SEM, are depicted in Figure 4C. The obtained micrographs revealed a decrease in filament spreading, resulting in a higher shape fidelity with the increasing content of NFC, which is in line with the Pr values obtained for the 3D structures as well as with the rheological data. From the 80:20 hydrogel, a fibrous surface morphology becomes more evident, clearly confirming the presence of NFC. Shin et al.^41^ also observed a similar fibrous morphology in GelMA/NFC inks (with 2% w/v of NFC), which was attributed to NFC.^41^

The presence of pores on 3D printed objects is critical because it allows cells to remain alive and proliferate while also promoting oxygen, nutrition, and waste material exchange.^65–67^ The porosity percentage of the 3D printed structures, evaluated using ethanol and determined by eq 7, tended to decrease with an increasing content of NFC, viz, 71.1 ± 7.9%, 75.9 ± 14.7%, 67.5 ± 12.9%, and 53.9 ± 20.6%, for 90:10, 80:20, 70:30, and 60:40, respectively; however, the differences between these results are not considered statistically significant. Nonetheless, these results confirm the existence of a porous network that cells would benefit from.

The ink 70:30 was chosen to print more complex structures (as shown in Figure 4D) as it has the best rheological properties and does not cause nozzle clogging while printing, which is frequently observed with the formulation 60:40. It was also possible to print a round grid-like structure (diameter 1.5 cm and height 5 mm) that is able to recover its initial shape after being manually compressed with a spatula, as illustrated in Figure 4E. The ink formulations developed in this work allowed for the 3D printing of Gel/NFC constructs with better resolution than the ones reported in the literature, as visually perceived in the works of Bedell et al.,^40^ Radeke et al.,^42^ and Luo et al.,^43^ for GelMA/NFC-based, cNFC/Gel-based, and Gel-alginate-NFC-based inks, respectively.

### 3.3 In Vitro Cytocompatibility of the Gel/NFC Hydrogels

Before cell-loading, it is essential to ensure that the hydrogel-based inks designed for 3D bioprinting applications do not cause harm to the selected cell line.^68^ Therefore, the in vitro cytotoxicity of the cross-linked Gel/NFC hydrogels toward HepG2 cells was evaluated via the MTT assay for periods of 24 and 48 h. Cell viabilities of all Gel/NFC formulations were well above the 70% cell viability threshold defined by ISO 10993-5:2009 for both time points, confirming that these formulations can be considered noncytotoxic.^69^ As shown in Figure 5A, 24 h after exposure, cell viabilities of 93 ± 3% and 93 ± 5% were obtained for the hydrogels 90:10 and 80:20, respectively, and slightly lower for those with compositions 70:30 and 60:40, with values of 89 ± 5% and 86 ± 4%, respectively. It is worth mentioning that the cell viabilities remained high even after 48 h of exposure, that is, 90 ± 6%, 88 ± 5%, 87 ± 5%, and 87 ± 4%, for 90:10, 80:20, 70:30, and 60:40, respectively. The obtained results are in accordance with the noncytotoxic nature of NFC,^70^ Gel,^71^ and even genipin^72^ toward the HepG2 cell line and highlight the potential of Gel/NFC inks to be laden with HepG2 cells for 3D bioprinting purposes.

**Figure 5:**
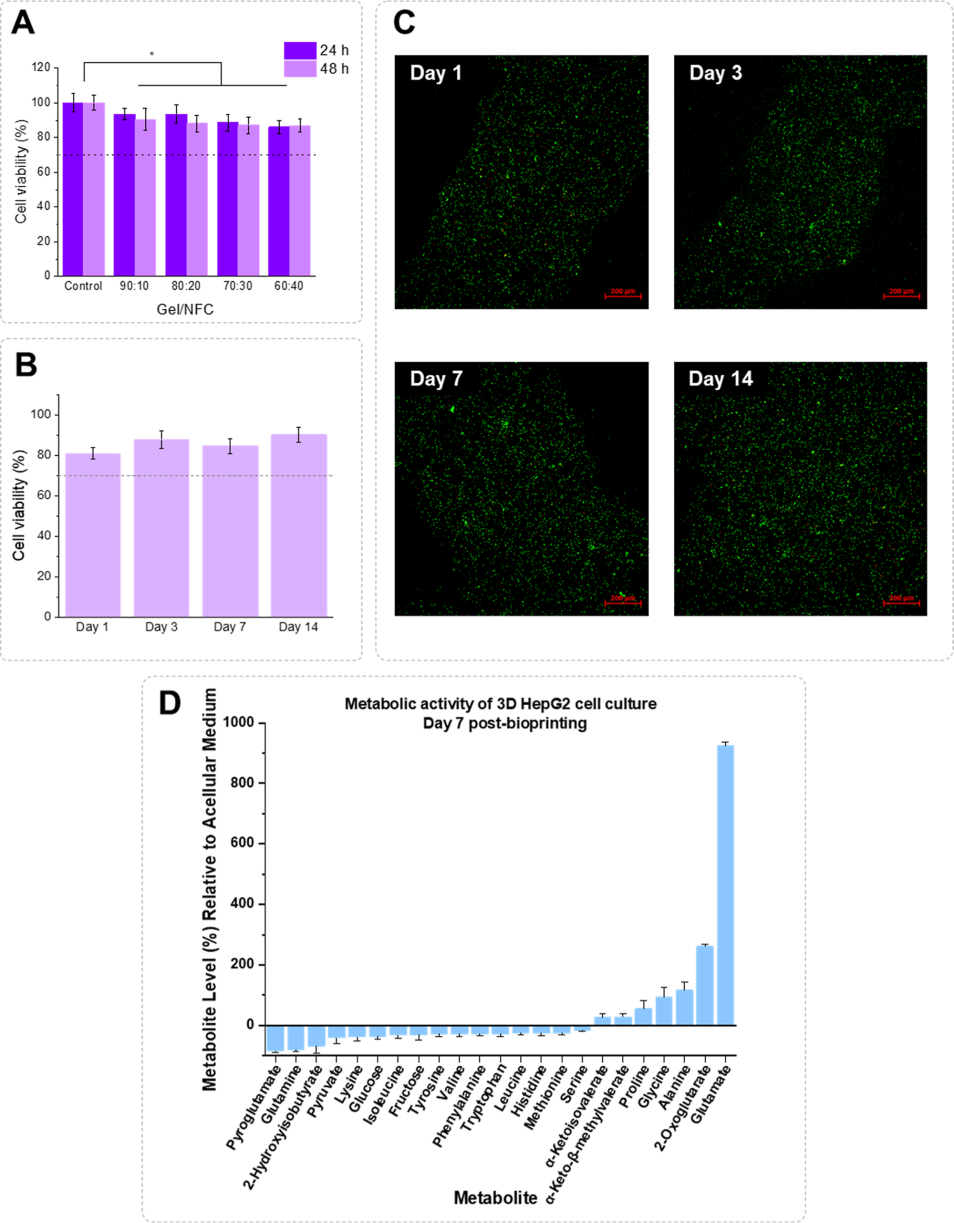
(A) Cell viability of HepG2 cells after 24 and 48 h of exposure to Gel/NFC hydrogels. The values are the mean of 6 replicates, and the bars correspond to the standard deviation (*p* < 0.05:*). (B) Cell viability at 1, 3, 7, and 14 days after bioprinting of the 70:30-based bioink. The represented values correspond to the mean of 3 measurements, and the error bars are the respective standard deviation (*p* < 0.05:*). (C) LIVE/DEAD fluorescence micrographs of the HepG2 cells encapsulated within the 70:30 hydrogel after 1, 3, 7, and 14 days postbioprinting. (D) Metabolite levels in the bioprinted HepG2-laden hydrogels medium, cultured for 7 days, expressed in percentage of variation relative to acellular medium. Metabolites that were consumed present negative variations, whereas metabolites secreted display positive variations. Bars represent the average variation ± percent error of three replicates.

### 3.4 3D Bioprinting of Gel/NFC Bioinks

The bioprinting of the cell-laden bioinks is the last step in verifying the feasibility of a designed formulation for obtaining 3D bioprinted models of living cells. The 3D bioprinting process may cause some stress on cells during extrusion of the material through the nozzle, normally resulting in a reduction of cell viability after bioprinting. Thus, bioinks play an important role in protecting cells from the shear stress and maintaining a high viability in the postbioprinting stage.^73^ The Gel/NFC ink with a weight ratio of 70:30 was chosen to be loaded with HepG2 cells (2 × 10^6^ cells mL^–1^), given its enhanced properties, namely, the rheological and mechanical features, capacity of maintaining its postprinting structure, degradability, and printability, as well as noncytotoxicity toward the chosen cell line. Additionally, and as previously reported (Section 3.2), the ink 70:30 was chosen to be loaded with cells due to its capacity of being smoothly extruded through the printing nozzle without clogging, which often happened with the 60:40 ink. The bioprinting of this bioink was carried out using the previously optimized printing parameters (0.41 mm, 2 bar, and 10 mm s^–1^) and the grid-like structures (20 × 20 × 0.320 mm, 1.6 mm filament distance, and 2 layers). The LIVE/DEAD test was used to determine the viability of the HepG2 cell line on days 1, 3, 7, and 14 postbioprinting. One day after the bioprinting, cell viability was 81 ± 3% and remained high throughout the 14 days of incubation, without significant differences, with cell viabilities reaching 88 ± 4%, 85 ± 4%, and 90 ± 4% at days 3, 7, and 14, respectively, as perceived in Figure 5B. These results reflect the positive impact of the shear-thinning behavior of the 70:30 ink, which promotes smooth extrusion of the bioink through the nozzle during bioprinting as it helps to shield the cells from the high shear rates felt during the bioprinting process. The high cell viability also highlights that the developed bioink is able to create an adequate environment for cell growth. Sun et al. also reported an increase in HepG2 cell viability throughout the 10 days postbioprinting (around 90%) for alginate-Gel-based bioinks.^15^ However, in a different study, Fritschen et al. reported high cell viabilities (above 80%), just during the first 3 days of culture, for a collagen-gelatin-based bioink.^74^ After that, cell viability drops below 60%, probably as a response to an increase in the aggregation of cells within the hydrogel, according to the authors.^74^ The clear prevalence of live cells (labeled in green) in the microscopy images shown in Figure 5C corroborates these results while simultaneously showing that the cells were homogeneously distributed within the bioprinted structure at all time points.

### 3.5 Metabolic Evaluation of the 3D Bioprinted HepG2 Cells

The bioink should be able to protect cells from stress during the bioprinting process while also being able to create an adequate environment for normal cell metabolic activity in the postbioprinting stage, which will translate into cell growth and proliferation. With this in mind, the metabolic activity of the HepG2 cells within the 3D structures was studied through the ^1^H NMR spectroscopic analysis of the medium supernatants. Relative metabolite levels in cells-conditioned medium, as assessed by spectral integration, were compared to those in acellular medium to determine which metabolites were consumed and secreted by cells during the 7 days of culture. The results are shown in Figure 5D. HepG2 cells in a 3D culture consumed about 80% of glutamine and pyroglutamate, together with 67% of 2-hydroxyisobutyrate, 40% of pyruvate, 35% of glucose, and 30% of fructose. Moreover, several amino acids were consumed in variable extents, between 17% (serine) and 38% (lysine). On the other hand, HepG2 cells secreted branched chain α-ketoacids as well as the amino acids proline, glycine, and alanine, along with 2-oxoglutarate and glutamate.

Interestingly, lactate, which is a typical metabolic product of cancer cells via the Warburg effect,^75^ was not secreted, and its levels remained similar to those in acellular medium. One hypothesis to explain this observation is that lactate derived from glycolysis is used as an anaplerotic substrate. Indeed, it has been demonstrated that HepG2 cells and HepG2 mitochondria take up lactate and convert it to pyruvate in a reaction catalyzed by a mitochondrial lactate dehydrogenase (mL-LDH).^76^ This was found to be preferred over direct pyruvate entry into the mitochondria of HepG2 cells and to help sustain the TCA cycle. Glutamine is another key substrate of HepG2 cells mitochondrial respiration.^77^ Accordingly, we have observed high consumption of glutamine and pyroglutamate, which are converted to glutamate and then to the TCA cycle intermediate 2-oxoglutarate (2-OG). Additionally, some glutamate, as well as 2-OG and alanine, was secreted, suggesting high activity of glutamate pyruvate transaminases, in agreement with previous studies on HepG2 cells.^77^

Altogether, our metabolic profiling results show that the bioprinted HepG2 cells maintain active metabolic pathways, greatly relying on mitochondrial oxidative metabolism. Previous studies have shown that HepG2 cells strongly depend on oxidative phosphorylation (OXPHOS) for energy production and survival, especially in response to different stress factors, such as low glucose availability,^78^ hypoxic stress,^79^ and chemotherapy drugs.^80^ Hence, the HepG2-laden 3D structures hereby produced represent a valuable tool for in vitro modeling of the in vivo microenvironment, where cells are challenged to adapt to dynamic and stressful conditions.

## 4 Conclusions

The current work addressed the development of a Gel/NFC hydrogel-based bioink with superior properties for 3D bioprinting of HepG2 cells using only genipin as the cross-linking agent. The addition of NFC to the formulations in different mass proportions, namely, 90:10, 80:20, 70:30, and 60:40 (Gel/NFC), contributed to the shear-thinning behavior, fundamental for the successful extrusion during the bioprinting process, which resulted in printed structures with better shape fidelity and resolution. Additionally, the mechanical properties and the stability (in DMEM and PBS) of the cross-linked Gel/NFC hydrogels were improved with the increasing content of NFC. Moreover, the hydrogels were nontoxic toward the HepG2 cell line, showing high cell viabilities for all hydrogels (consistently above 80%), at the selected points. The ink with the best combination of properties (70:30) was loaded with HepG2 cells and bioprinted. The fluorescence micrographs showed that cells were evenly distributed within the bioprinted structures, and cell viability remained considerably high throughout the 14 days (up to 90 ± 4%) of incubation. Furthermore, metabolic evaluation at day 7 after bioprinting revealed that HepG2 cells retained several typical characteristics of the metabolic behavior of these cells, evidencing that the Gel/NFC 3D structures effectively allowed HepG2 cells to stay alive and maintain high metabolic activity postbioprinting.

In summary, this work demonstrated the possibility of creating a Gel/NFC bioink (with a simple cross-linking mechanism, using genipin) with superior performance (viz, rheological, mechanical, stability, printability, and noncytotoxicity) that successfully acted as a supporting matrix for HepG2 cells during the bioprinting process and postbioprinting stage. Thus, the formulated bioink represents a new approach for bioprinting HepG2 cells, particularly for biomedical applications.

## Abbreviations

^1^H NMR: proton nuclear magnetic resonance
2-OG: 2-oxoglutarate
D_2_O: deuterium oxide
DMEM: Dulbecco’s Modified Eagle’s Medium
DMSO: dimethyl sulfoxide
FBS: fetal bovine serum
Gel: Gelatin
GelMA: gelatin methacrylate
HepG2: Hepatocellular carcinoma cells
mL-LDH: mitochondrial lactate dehydrogenase
MTT: 9-(4,5-dimethylthiazolyl-2)-2,5-diphenyltetrazolium bromide
NFC: nanofibrillated cellulose
OXPHOS: oxidative phosphorylation
PBS: phosphate buffer saline
RGD: arginine-glycine-aspartate
SEM: scanning electron microscopy
TCA: tricarboxylic acid
TSP-*d*_4_: 3-(trimethylsilyl) propionic acid
UV: ultraviolet.
